# Loss of Neuropilin-2 in Murine Mesenchymal-like Colon Cancer Organoids Causes Mesenchymal-to-Epithelial Transition and an Acquired Dependency on Insulin-Receptor Signaling and Autophagy

**DOI:** 10.3390/cancers14030671

**Published:** 2022-01-28

**Authors:** Susanna Poghosyan, Nicola Frenkel, Aristeidis Lentzas, Jamila Laoukili, Inne Borel Rinkes, Onno Kranenburg, Jeroen Hagendoorn

**Affiliations:** Laboratory for Translational Oncology, Department of Surgical Oncology, Division of Imaging and Cancer, University Medical Center Utrecht, Utrecht University, Heidelberglaan 100, 3584 CX Utrecht, The Netherlands; S.Poghosyan@umcutrecht.nl (S.P.); N.C.Frenkel-3@umcutrecht.nl (N.F.); a.lentzas@nki.nl (A.L.); J.Laoukili@umcutrecht.nl (J.L.); i.h.m.borelrinkes@umcutrecht.nl (I.B.R.); o.kranenburg@umcutrecht.nl (O.K.)

**Keywords:** organoids, colorectal cancer, Neuropilin-2, autophagy, insulin receptor, mesenchymal-to-epithelial transition (MET)

## Abstract

**Simple Summary:**

Many cancer types are reported to have high lymphangiogenic receptor Neuropilin-2 (Nrp2) expression, including colorectal cancer (CRC). Nrp2 is shown to be associated with tumor progression in vivo and poor prognosis in CRC patients. Although the role of Nrp2 is well established in lymphangiogenesis, the tumor cell-intrinsic role of Nrp2 remains elusive. Here, we employed murine CRC tumor-derived mesenchymal-like organoids to induce Nrp2 depletion. We demonstrate that Nrp2 deletion in CRC organoids results in a drastically altered phenotype that is characterized by mesenchymal-to-epithelial transition (MET), and an acquired dependency on IR signaling and autophagy. This phenotype is preserved in subcutaneous tumors generated by CRC organoids. We conclude that there is a complex interaction between Nrp2 and alternative pro-survival mechanisms in aggressive CRC, which could be therapeutically exploited.

**Abstract:**

Neuropilin-2 (Nrp2), an important regulator of lymphangiogenesis and lymphatic metastasis, has been associated with progression in colorectal cancer (CRC). However, the tumor cell-intrinsic role of Nrp2 in cancer progression is incompletely understood. To address this question, we employed CRISPR-Cas9 technology to generate Nrp2-knockout organoids derived from murine CRC tumors with a mesenchymal phenotype. Transcriptome profiling and tumor tissue analysis showed that Nrp2 loss resulted in mesenchymal-to-epithelial transition (MET), which was accompanied with restored polarity and tight junction stabilization. Signaling pathway analysis revealed that Nrp2-knockout organoids acquire de novo dependency on insulin receptor (IR) signaling and autophagy as alternative survival mechanisms. Combined inhibition of IR signaling and autophagy prevented the stabilization of cell-cell junctions, reduced metabolic activity, and caused profound cell death in Nrp2-knockout organoids. Collectively, the data demonstrate a key role for Nrp2 in maintaining the aggressive phenotype and survival of tumor-derived CRC organoids. The identified connection between Nrp2, insulin receptor signaling and autophagy may guide the development of novel combination-treatment strategies for aggressive CRC.

## 1. Introduction

Colorectal cancer (CRC) has a considerably high mortality rate due to the development of distant metastases. Colorectal liver metastases (CRLM) develop in 25–50% of CRC patients [1,2,3]. Despite advances in surgical procedures and systemic therapy, therapeutic efficacy, and patient prognosis remain poor [4]. CRC is a heterogeneous disease, which can be classified into four distinct consensus molecular subtypes (CMS), based on gene expression profiles, reflecting marked differences in the activation of specific signaling pathways [5]. CMS4 subtype is characterized by the high expression of genes reflecting epithelial-to-mesenchymal transition (EMT), transforming growth factor (TGF)-β signaling, and high stromal cell content. CMS4 is associated with a significantly worse disease-free and overall survival [5].

We previously reported that high lymphangiogenic gene expression, including Neuropilin (Nrp)-2, is predictive of CMS4 and a poor prognosis in both primary CRC and CRLM [6]. Lymphangiogenesis plays a key role in the progression of solid tumors, such as CRC [7,8,9,10]. Nrp2 is a key regulator of lymphangiogenesis, including tumor lymphangiogenesis [11,12]. It can be expressed in both lymphatic endothelial cells (LECs) and cancer cells. As an independent receptor or a co-receptor, Nrp2 binds to ligands VEGF-C/D, activates the VEGF-C/D-Nrp2 signaling axis, and further regulates lymphangiogenesis-associated factors in both lymphatic endothelial cells (LECs) and tumor cells [13,14]. Despite being upregulated in tumor-associated lymphatic vessels, Nrp2 is also highly expressed in several cancer types. It was shown to modulate various cellular pathways, such as angiogenesis, cellular communication, migration, and survival [14,15,16,17,18,19]. It also acts as a functional receptor for semaphorin-3F, which mediates p53-regulated tumor angiogenesis suppression [20]. In vitro work using CRC cell lines has shown that Nrp2 can induce proliferation and EMT by cooperating with the TGFβ1 receptor, and restrict apoptosis to promote cancer progression [21,22,23]. Conversely, depletion of Nrp2 in tumors reduces growth, survival under hypoxic conditions, and invasiveness [24,25], while in vivo targeting of Nrp2 reduces the growth of CRC cells implanted in the liver [25].

Despite increasing evidence of the tumoral Nrp2 association with CRC progression, mechanisms underlying Nrp2-mediated colorectal cancer growth and metastasis remain elusive. To study the tumor cell-intrinsic role of Nrp2 in the aggressive form of CRC, we aimed to generate a CRC model closely resembling the clinical correlate, EMT-high/stroma-rich CMS4 subtype. Recently developed three-dimensional (3D) organoid culture technology allows for the conservation of genetic heterogeneity and phenotypic characteristics of malignant cells, as in the initial cancer tissue [26,27,28]. We used Notch1-activated/p53-deleted tumors to generate mesenchymal-like CRC organoids. Tumors recapitulated the mesenchymal/EMT-like phenotype of invasive human CRC and were obtained from a spontaneously metastasizing transgenic mouse model generated by the conditional activation of the Notch1 receptor and p53 deletion in the digestive epithelium [29]. Generated organoids were used to establish a novel CRC organoid model depleted of Nrp2 expression using CRISPR-Cas9 technology. Nrp2-knockout (Nrp2^−/−^) mesenchymal-like CRC organoids were employed in vitro 3D and in vivo subcutaneous tumorigenesis models to explore the mechanisms underlying the cancer cell-intrinsic pro-tumorigenic effects of Nrp2.

## 2. Materials and Methods

### 2.1. In Vitro 3D Organoid Culture and Treatment

In this study, we used Notch1-activated/p53-deleted model-derived tumor (*n* = 4) organoids. Tumors were generated in a spontaneously metastasizing murine CRC model [29]. Organoids were established as previously described [30]. For passaging, organoids were dissociated with TrypLE Express mix (Gibco, 12604021) for 5 min in a 37 °C water bath. Organoids were cultured by embedding them in ice-cold Matrigel (Corning, 356231). Organoids in murine CRC culture medium (Table S4) were mixed with ice-cold Matrigel in a 1:3 ratio and plated as 10 µL droplets in pre-warmed adherent cell culture plates. Plates were inverted and placed at 37 °C for droplets to solidify, followed by a murine CRC culture medium addition. The medium was refreshed every two days.

Prior to the treatment, three days old organoids were dissociated into single cells by using TrypLE Express and incubating for 5 min in a 37 °C water bath. To analyze insulin receptor activation, cells were treated with 10 µg/mL of insulin (Sigma, I9278, St. Louis, MO, USA) for 2 min, 5 min, and 10 min. Next, cells were lysed using RIPA buffer for Western blot analysis.

For immunofluorescence analysis, three days old organoids were treated with 30 µM linsitinib (Selleckchem, OSI-906, Houston, TX, USA), Chloroquine (Invivogen, tlrl-chq, San Diego, CA, USA) or Spautin-1 (Sigma, SML0440) for 24 h at 37 °C.

### 2.2. RNA Sequencing and Data Analysis

For transcriptome profiling of two Nrp2^−/−^ CRC organoid lines, total RNA was isolated from three days old organoids using Qiagen RNeasy kit (74104) according to the manufacturer’s instructions. For each organoid line, three RNA replicates were used for sequencing, isolated from three different organoid culture passages. First, organoids were treated with 2 mg/mL Dispase II (Gibco, 17105041) for 15 min at 37 °C to disrupt Matrigel droplets. Organoids were lysed using an RLT RNA lysis buffer. Isolated total RNA quality was checked using the Agilent RNA 6000 Nano kit (5067-1511) and Agilent 2100 Bioanalyzer. RNA samples with high integrity numbers (RIN 9–10) were used for sequencing. Sequencing was performed by the Utrecht Sequencing Facility using the Nextseq500 platform, high output 1 × 75 bp run type. RNA expression data were analyzed using the R2: Genomics Analysis and Visualization Platform (http://r2.amc.nl (accessed on 24 May 2019).). The Nrp2^−/−^ CRC organoid RNA sequencing dataset is available on the platform as Exp aM-L2 mNRP2_KO-Kranenburg-15-custom-mm10ens79. The human CRC RNA sequencing dataset is available as Tumor Colon CIT (Combat)-Marisa-566-rma-u133p2.

### 2.3. In Vivo Subcutaneous Tumorigenesis

Healthy 8–10 week old 25–30 g male C57Bl/6 mice were supplied by Charles River. Animals were randomly allocated in groups of 3 mice into Type II cages with a filter top. Animals were kept at room temperature under 12 h light/dark cycles and received standard chow pellets and water ad libitum. The research was approved by the Competent Authority, The Netherlands (License number AVD115002016614), which is advised by the Animal Ethics Committee. Animal work protocols were approved by the Animal Welfare Body and were performed in accordance with the Dutch Law on Animal Experiments and the European Directive 2010/63/EU.

The three days old, control, and Nrp2^−/−^ hepatic CRC organoids were treated with 2 mg/mL Dispase II (Gibco, 17105041) for 15 min at 37 °C to disrupt the Matrigel droplets. Next, organoids were dissociated into single cells by using TrypLE Express (Gibco, 12604021) for 5 min in a 37 °C water bath. Cell suspension in HBSS and Matrigel (Corning, 356231) was prepared in a 1:1 ratio. Furthermore, 5 × 105 cells in 100 µL volume were injected into the right flank of animals using a 1 ml syringe and 25 G 0.5 × 16 mm needles. Animal welfare was monitored by physical appearance, behavior, and body weight. Tumor size was measured every two days post-injection by using a digital caliper. Tumor volume (V) was estimated as V = 1/2 × (smaller diameter^2^ × larger diameter). Animals were sacrificed at twelve weeks post-injection. Tumor tissue was harvested for further immunohistochemical analyzes.

### 2.4. Immunohistochemistry (IHC) and Immunofluorescence (IF)

Tumor tissue was fixed in 4% formaldehyde solution and paraffin-embedded. Sections of 4 µm thickness were made. Prior to the staining, tissue sections were deparaffinized and rehydrated. Endogenous peroxidase activity was blocked with 1.5% hydrogen peroxide for 30 min. Heat-induced antigen retrieval was carried out using citrate buffer pH6.0 for 20 min, followed by the cooling of tissue sections for 20 min. To prevent non-specific binding, tissue sections were treated with UltraVision protein block (Fisher Scientific, TA-125-PBQ, Waltham, MA, USA) for 5 min. Sections were incubated with primary antibodies (Table S5) overnight at 4 °C, followed by a 1 h incubation with the HRP-conjugated secondary antibody. Between antibody incubations, sections were washed twice with 0.05% Tween-PBS solution for 5 min and once with 1× PBS for 5 min. Sections were developed with 3,3’-Diaminobenzidine (DAB) chromogen for 10 min at room temperature in the dark. Sections were rinsed under running tap water for 10 min and counterstained with hematoxylin, followed by dehydration and mounting. Furthermore, 20× or 40× magnification images were acquired using the Nikon Eclipse E800 microscope. Mean DAB intensity was quantified relative to the control.

For immunofluorescence analysis, organoids were cultured on 15 well µ-slides (ibidi, 81506) for three to five days. The medium was removed from the wells, and organoids were fixed with 4% formaldehyde solution for 1.5 h at room temperature. The reaction was quenched using 0.05 M NH4Cl for 30 min at room temperature. Next, the wells were washed twice with the assay buffer containing 1× PBS with 10% Triton, 1% DMSO, 1% BSA. Organoids were stained with primary antibodies (Table S5) diluted in the assay buffer overnight at 4 °C. Organoids were incubated with secondary antibodies (Table S5) in the assay buffer in the dark. After washing three times with the assay buffer for 5 min, 1 drop of mounting medium (ibidi, 50011) was added to each µ-slide well. Organoids were imaged with the Zeiss LSM700 confocal microscope using Zeiss ZEN 2011 software. Images were acquired at 63× magnification with oil immersion. Mean fluorescence intensity was quantified relative to the control.

### 2.5. Western Blot Analysis

Three to five days old CRC organoids were treated with 2 mg/mL Dispase II (Gibco, 17105041) for 15 min at 37 °C to dissolve Matrigel. After washing with PBS, organoids were lysed using Laemmli buffer (10% glycerol, 2% SDS, 63 mM Tris-HCl pH6.8). A total protein concentration was determined using the Lowry method [31]. Proteins were separated via SDS-PAGE gel-electrophoresis, and transferred on 0.2 µm PVDF membranes using a Trans-blot Turbo transfer system (BioRad, Hercules, CA, USA). PageRuler plus prestained protein ladder (ThermoFisher, 26619, Waltham, MA, USA) was used. After 1 h blocking of unspecific binding with 5% non-fat dry milk in 1× TBS-0.1% Tween solution, membranes were incubated with primary antibodies overnight at 4 °C. Next, membranes were incubated with HRP-conjugated secondary antibodies for 1 h at room temperature. Antibodies (Table S5) were diluted in 1% non-fat dry milk 1× TBS-0.1% Tween solution or 1% BSA 1× TBS-0.1% Tween solution (for detection of phosphoproteins). After each antibody incubation, membranes were washed three times with 1× TBS for 5 min. Protein detection was performed using enhanced chemiluminescence (ECL) reagents (Amersham ECL, GE Healthcare, RPN2235/2209, Chicago, IL, USA). For quantification, the protein signal was measured in ImageJ using the ROI manager tool. The signal was corrected for protein loading differences using either β-actin or α-tubulin and quantified relative to the control. The whole western blot figures can be found in the supplementary materials.

### 2.6. Statistical Analyzes

The analyzes were performed using one-way ANOVA, and *p*-values of ≤0.05 were considered significant. Dunnett’s test was applied to correct for multiple comparisons. Error bars on graphs indicate the standard deviation from the mean of independent measurements. Statistical analysis and graphs were made using GraphPad Prism 8.0 (GraphPad Software, LaJolla, CA, USA). Figures were prepared in Adobe Illustrator, Version 24.3.

## 3. Results

### 3.1. Nrp2 Knockout in Mouse CRC Organoids Induces Tight Junction Stabilization and EMT Reduction

**Figure 1:**
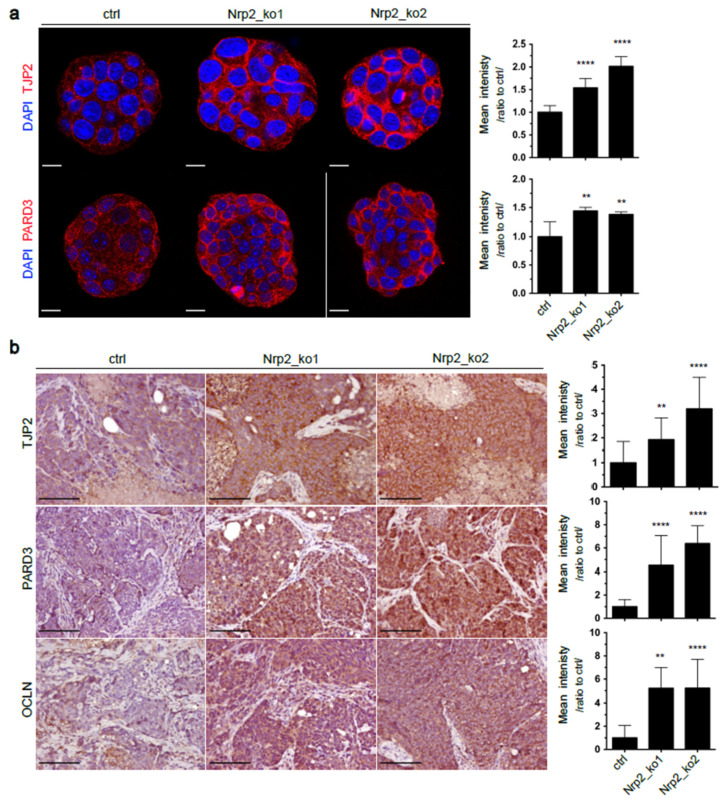
Increased tight junction protein expression in Nrp2^−/−^ CRC organoids. (**a**) Immunofluorescence analysis showing TJP2 and PARD3 expression in CRC organoids. Representative organoid images are depicted, where DAPI-stained nuclei are shown in blue, and membranous TJP2 and PARD3 are shown in red. Mean fluorescence intensity is quantified relative to the control (*n* = 5). Scale bars: 15 µm. (**b**) Immunohistochemical analysis of subcutaneous tumor tissues generated by the control or Nrp2^−/−^ CRC organoids. TJP2, PARD3, and OCLN expression were quantified as mean DAB area intensity relative to the control (*n* = 3). Scale bars: 100 µm. Error bars are presented as SD from the arithmetic mean. Statistical significance on graphs is indicated *p* ≤ 0.01 by **, *p* ≤ 0.0001 by ****.

**Figure 2:**
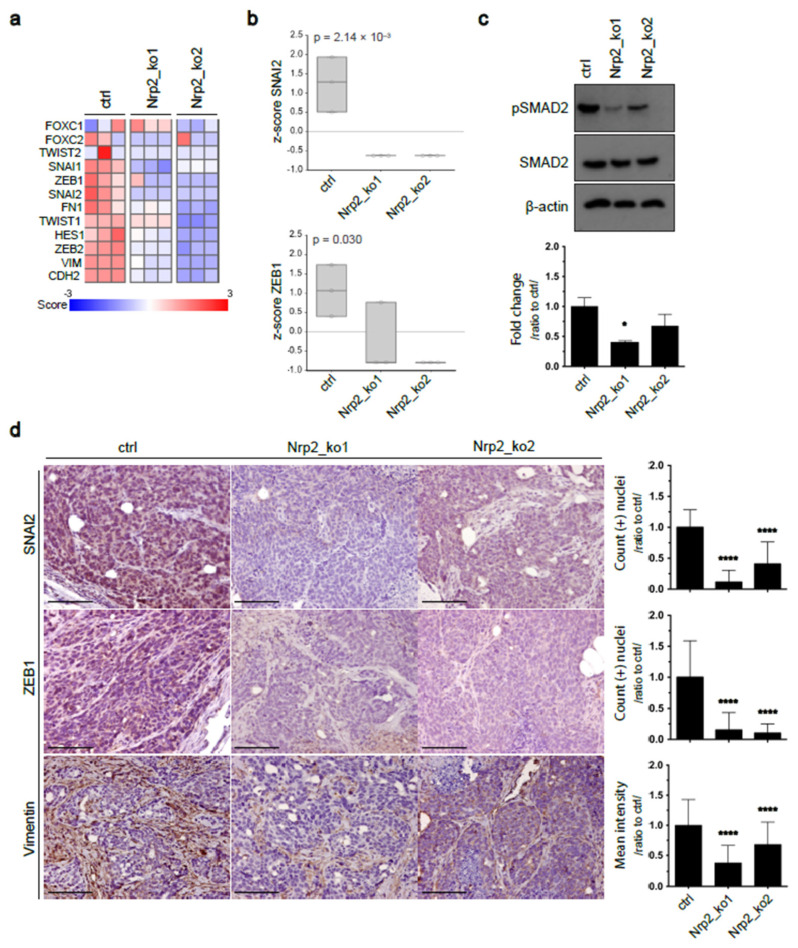
Nrp2 knockout causes mesenchymal-to-epithelial transition (MET) in CRC organoids. (**a**,**b**) RNAseq data analysis for EMT marker expression in CRC organoids. Samples were sequenced in three replicates. (**c**) Western blot analyzes of SMAD2 and phospho-SMAD2 expression in CRC organoids. Phospho-SMAD2 expression was quantified relative to SMAD2 and β-actin and normalized to the control (*n* = 2). (**d**) Immunohistochemical analysis of subcutaneous tumor tissues generated by the control or Nrp2^−/−^ CRC organoids. EMT marker SNAI2 and ZEB1 expression were quantified based on DAB-positive nuclei count, and vimentin expression was quantified as mean DAB area intensity, relative to the control (*n* = 3). Scale bars: 100 µm. Error bars are presented as SD from the arithmetic mean. Statistical significance on graphs is indicated *p* ≤ 0.05 by *, *p* ≤ 0.0001 by ****.

To study the tumor cell-intrinsic function of Nrp2, we applied CRISPR-Cas9 genome editing to establish Nrp2-depleted (Nrp2^−/−^) mouse CRC organoids (Supplementary file, Figure S1, Table S1–S3). Generated organoids were sequenced for differential RNA expression analysis, which revealed increased expression of tight junction proteins, such as PARD3 and TJP2, in Nrp2^−/−^ CRC organoids (Figures S2b and S3). Immunofluorescence analysis confirmed an increased expression of these markers in Nrp2^−/−^ CRC organoids (Figure 1a). Next, we explored the effect of Nrp2 knockout in vivo. Subcutaneous implantation of control and Nrp2^−/−^ organoids into mice resulted in tumor formation in all groups. The analysis of tumor tissue by immunohistochemistry showed a high expression of tight junction proteins PARD3, TJP2, and occludin (OCLN) in subcutaneous tumors formed by Nrp2^−/−^ CRC organoids (Figure 1b). In addition, Nrp2 knockout in CRC organoids resulted in a significant downregulation of core EMT-driving transcription factors, including ZEB1, ZEB2, SNAI1, and SNAI2. The expression of mesenchymal genes, such as vimentin (VIM) and N-cadherin (CDH2), was also strongly reduced in Nrp2^−/−^ organoids (Figure 2a,b). These results show that Nrp2 knockout leads to a mesenchymal-to-epithelial transition (MET). Nrp2 has been previously reported to induce TGFβ-mediated EMT in CRC cell lines [21]. In line with these observations, we show reduced basal levels of SMAD2 phosphorylation in Nrp2^−/−^ CRC organoids compared to the control organoids (Figure 2c), indicating that reduced TGFβ signaling may be, at least partially, responsible for the observed EMT to MET switch. 

The MET transition was also observed in subcutaneous tumors formed by Nrp2^−/−^ CRC organoids, which was demonstrated by a significant reduction of EMT drivers, ZEB1, and SNAI2, and a mesenchymal marker, vimentin (Figure 2d). Subcutaneous tumors generated by control CRC organoids displayed an extensive stromal infiltration, which was severely reduced upon Nrp2 knockout.

### 3.2. Nrp2 Knockout in Mouse CRC Organoids Reduces Adult Intestinal Stem Cell Gene Expression without Influencing Fetal Intestinal Stem Cell Signature

**Figure 3:**
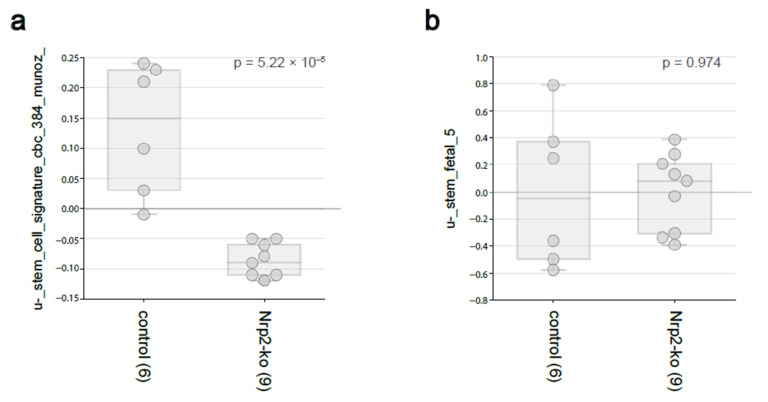
The stem-like phenotype in Nrp2^−/−^ CRC organoids. RNAseq data analysis for (**a**) adult Lgr5+ intestinal stem cell and (**b**) fetal intestinal stem cell gene-signature expression in mouse CRC organoids using R2: Genomics Analysis and Visualization Platform.

To investigate the effect of Nrp2 loss on stem-like phenotype of CRC organoids, we have analyzed the expression of adult [32] and fetal [33] intestinal stem cell gene signatures according to the Nrp2 status. These analyzes revealed that the signature derived from adult Lgr5+ intestinal stem cells was strongly and significantly reduced following Nrp2 knockout (Figure 3a), whereas the expression of a fetal intestinal stem cell signature was unaltered (Figure 3b). These data reinforce the link between cancer stemness, reflected by the Lgr5 signature, and EMT in colon cancer and identify Nrp2 as an important factor in maintaining this phenotype.

### 3.3. Nrp2 Knockout in Mouse CRC Organoids Induces Insulin Receptor Signaling Upregulation

**Figure 4:**
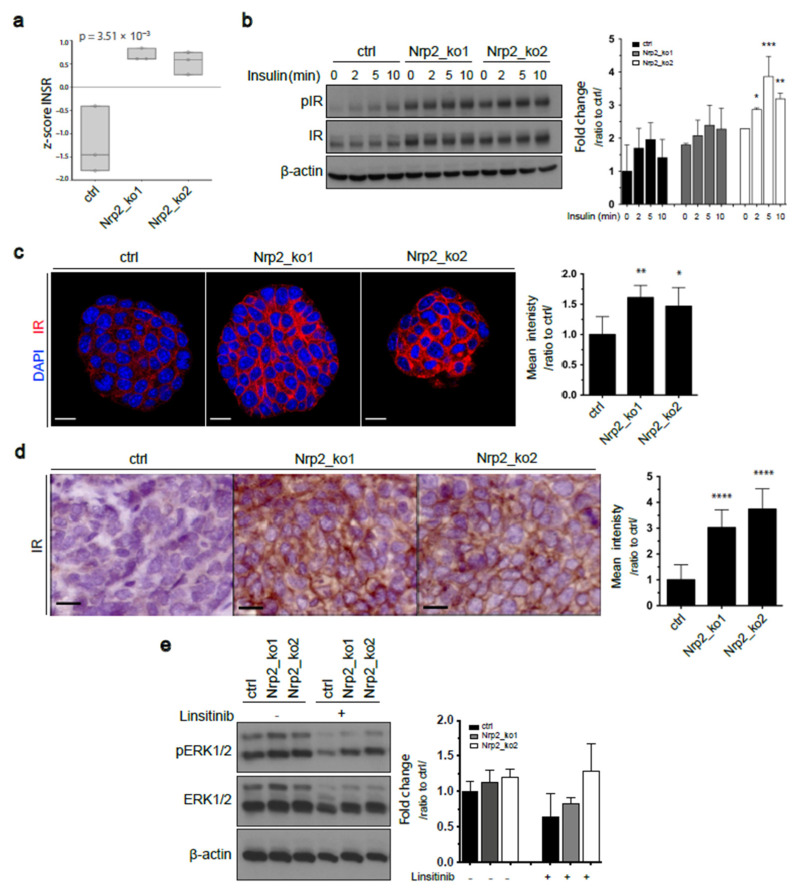
Increased insulin receptor signaling in Nrp2^−/−^ CRC organoids. (**a**) Insulin receptor (IR) RNA levels in control and Nrp2^−/−^ organoids measured by RNA sequencing. Samples were sequenced in three replicates. (**b**) Western blot analysis of IR and phospho-IR (Tyr1146) levels in the control and Nrp2^−/−^ CRC organoids following stimulation with 10µg/mL insulin. Phospho-IR expression was quantified relative to IR and β-actin and normalized to unstimulated control (*n* = 2). (**c**) Immunofluorescence analysis showing IR expression in CRC organoids. Representative organoid images are depicted, where DAPI-stained nuclei are shown in blue, and membranous IR is shown in red. Mean fluorescence intensity is quantified relative to the control (*n* = 5). Scale bars: 15 µm. (**d**) Immunohistochemical analysis of IR expression in subcutaneous tumor tissues generated by the control or Nrp2^−/−^ CRC organoids. Mean DAB area intensity is quantified relative to the control (*n* = 3). (**e**) Western blot analyzes of ERK1/2 and phospho-ERK1/2 expression in untreated or 30 µM IR inhibitor (Linsitinib)-treated CRC organoids. Phospho-ERK1/2 expression was quantified relative to ERK1/2 and β-actin and normalized to the untreated control (*n* = 2). Scale bars: 15 µm. Error bars are presented as SD from the arithmetic mean. Statistical significance on graphs is indicated *p* ≤ 0.05 by *, *p* ≤ 0.01 by **, *p* ≤ 0.001 by ***, *p* ≤ 0.0001 by ****.

The expression profile of receptor tyrosine kinases (RTKs) in tumor cells provides key information on their oncogenic potential and possible molecular mechanisms involved in tumorigenesis. Differential gene expression analysis revealed increased RNA expression of key RTKs, and more significantly, insulin receptor (IR) expression in Nrp2-knockout organoids (Figure 4a and Figure S2a). Increased IR protein levels in Nrp2^−/−^ organoids were confirmed by Western blotting (Figure 4b). Immunofluorescence analysis showed that IR was expressed at the plasma membrane throughout Nrp2^−/−^ organoids, but not the control organoids (Figure 4c). Immunohistochemistry on subcutaneous tumor tissue sections showed an increased IR expression on the cell membrane of Nrp2^−/−^ tumors, as was observed in the organoids (Figure 4d).

Nrp2 has been reported to regulate epidermal growth factor receptor (EGFR) levels in cancer cells [34]. We demonstrate a significant reduction in EGFR expression upon Nrp2 knockout in CRC organoids (Figure S2a). However, this did not result in a reduction in ERK1/2 phosphorylation. Presumably, this is due to the activation of feedback pathways such as IR signaling. Treatment with the IR signaling inhibitor resulted in reduced ERK1/2 phosphorylation, and more considerably in the control CRC organoids (Figure 4e).

### 3.4. Nrp2 Depletion in Mouse CRC Organoids Activates Autophagy

**Figure 5:**
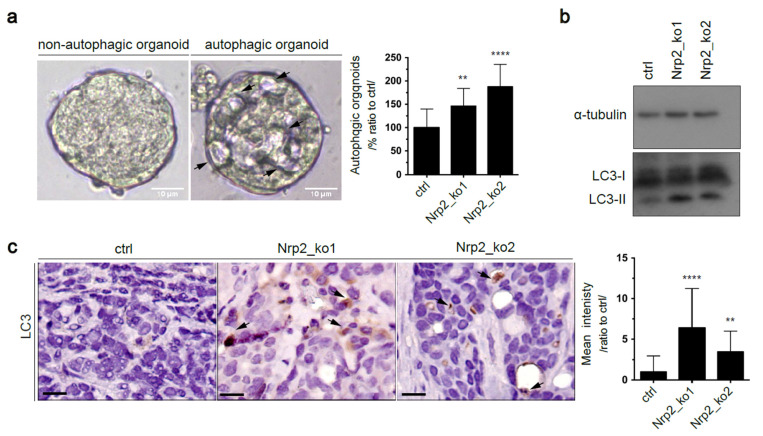
Nrp2 knockout induces autophagy. (**a**) Bright field images of control and Nrp2-knockout organoids. Arrows indicate vacuolar structures. Scale bars:10 µm. The number of organoids with vacuole-like structures was quantified relative to the control (*n* = 3). Five images per condition were analyzed in each experiment. (**b**) Western blot analysis showing LC3-II marker expression in CRC organoids. (**c**) Immunohistochemical analysis of subcutaneous tumor tissues generated by the control or Nrp2^−/−^ CRC organoids. LC3-II puncta accumulation was quantified as mean DAB area intensity relative to the control (*n* = 3). Scale bars: 15 µm. Error bars are presented as SD from the arithmetic mean. Statistical significance on graphs is indicated *p* ≤ 0.01 by ** and *p* ≤ 0.0001 by ****.

We observed that Nrp2^−/−^ CRC organoids displayed altered morphology. Structures resembling autophagic vacuoles were accumulated (Figure 5a). Autophagy is an essential mechanism for the maintenance of cellular homeostasis [35,36]. In cancer, it is suggested that autophagy may have a pro-tumor effect by supporting cell survival in conditions of hypoxia and nutrient deprivation. We performed Western blot analysis, which showed high levels of the autophagosomal marker LC3-II in Nrp2^−/−^ CRC organoids (Figure 5b). In addition, LC3 puncta accumulated in subcutaneous Nrp2^−/−^ CRC tumors (Figure 5c). Autophagosomal LC3-II accumulation can be indicative of autophagy induction or a blockade in autophagosome maturation [37,38,39]. To functionally show that autophagy was upregulated upon Nrp2 loss, we measured autophagic flux—the dynamic process of autophagy—using autophagy inhibitors. We treated CRC organoids with either an early autophagy inhibitor (Spautin-1), which inhibits autophagy initiation [40] or with a late autophagy inhibitor (chloroquine), which blocks autophagosome-lysosome fusion [41]. We found that treatment with autophagy inhibitors, particularly with the late inhibitor (chloroquine), induced relatively high levels of LC3-II in Nrp2^−/−^ CRC organoids compared to the control organoids (Figure S4b), thus indicating increased autophagy flux in Nrp2^−/−^ CRC organoids.

In addition, we measured autophagic flux by p62 degradation after autophagy inhibition; p62 is a cargo protein destined to be degraded in autophagy as it carries the ubiquitinated protein aggregates for autophagosomal degradation [42]. Autophagy suppression correlates with an increased p62 level [43]. Similar to LC3-II results, the treatment with autophagy inhibitors resulted in an increase in p62 levels in Nrp2^−/−^ CRC organoids (Figure S4a). Collectively, this data show autophagy induction upon Nrp2 depletion.

### 3.5. Combined Inhibition of IR Signaling and Early Autophagy Reduces Nrp2^−/−^ CRC Organoid Viability

**Figure 6:**
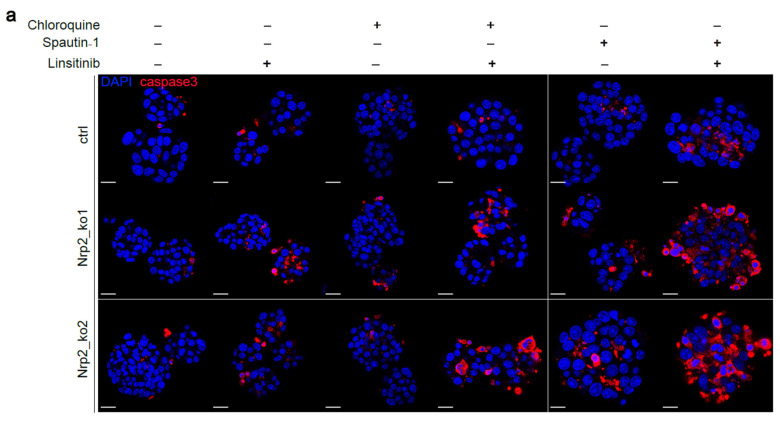

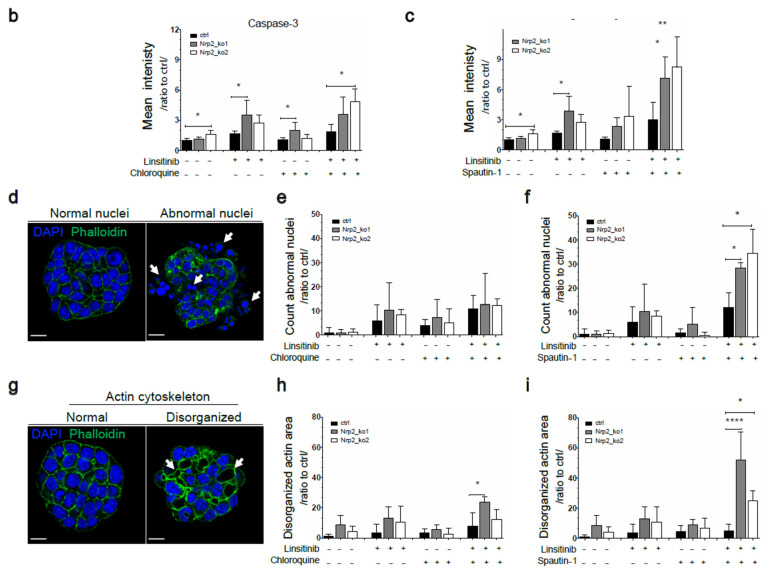
Nrp2 knockout sensitizes CRC organoids to combined inhibition of IR signaling and autophagy. Immunofluorescence analysis of CRC organoids after single or combination treatment with early autophagy inhibitor (Spautin-1), late autophagy inhibitor (Chloroquine), and IR signaling inhibitor (Linsitinib). Each inhibitor was used at 30 µM concentration for 24 h. (**a**) Apoptotic marker, cleaved caspase-3 expression analyzed in Nrp2^−/−^ CRC organoids. Representative organoid images are depicted, where DAPI-stained nuclei are shown in blue, and cleaved caspase-3 is shown in red. Mean fluorescence intensity was quantified relative to the untreated control (**b**) for the combination treatment with chloroquine and linsitinib (*n* = 5), and (**c**) for the combination treatment with Spautin-1 and linsitinib (*n* = 5). (**d**) Representative organoid images with normal and abnormal nuclei (white arrows). DAPI-stained nuclei are shown in blue, and actin phalloidin is shown in green. The number of abnormal nuclei was quantified relative to the untreated control (**e**) for the combination treatment with chloroquine and linsitinib (*n* = 5), and (**f**) for the combination treatment with Spautin-1 and linsitinib (*n* = 5). (**g**) Representative organoid images with the normal and disorganized cytoskeletion (white arrows). DAPI-stained nuclei are shown in blue, and actin phalloidin is shown in green. Disorganized cytoskeletion area was quantified relative to the untreated control (**h**) for the combination treatment with chloroquine and linsitinib (*n* = 5), and (**i**) for the combination treatment with Spautin-1 and linsitinib (*n* = 5). Scale bars: 15 µm. Error bars are presented as SD from the arithmetic mean. Statistical significance on graphs is indicated *p* ≤ 0.05 by *, *p* ≤ 0.01 by **, and *p* ≤ 0.0001 by ****.

Nrp2 knockout significantly reduced the regeneration capacity of Nrp2^−/−^ CRC organoids in a clone forming assay (Figure S5a). We hypothesized that the depletion of Nrp2 as an important cell survival receptor may result in dependency on alternative survival mechanisms, such as IR signaling and autophagy. Thus, suppression of these pathways should influence organoid viability. Indeed, IR inhibition resulted in the accumulation of cleaved (activated) caspase-3, an apoptotic marker (Figure 6a–c). Combined inhibition of IR signaling and autophagy, particularly the treatment with an early autophagy inhibitor, led to a drastic increase in cleaved caspase-3 levels. Morphological alterations, such as chromatin condensation, nuclear shrinkage, and membrane blebbing, are characteristic features of apoptosis [44,45]. We demonstrate that the combined inhibition of IR signaling and autophagy results in abnormal nuclei accumulation and cytoskeleton disorganization in Nrp2^−/−^ CRC organoids (Figure 6d–i).

**Figure 7:**
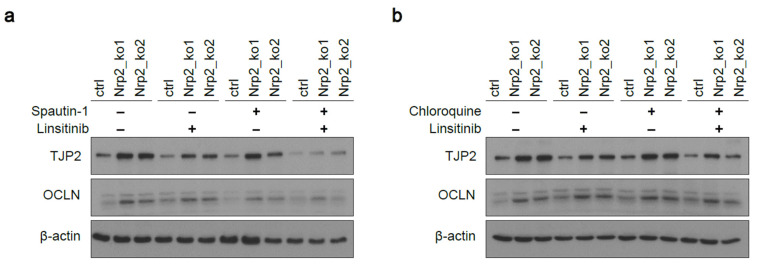
Combined inhibition of IR signaling and early autophagy reduces tight junction protein levels in Nrp2^−/−^ CRC organoids. Western blot analysis of TJP2 and OCLN expression after treating the CRC organoids (**a**) with early autophagy inhibitor (Spautin-1) and IR signaling inhibitor (linsitinib), and (**b**) with late autophagy inhibitor (chloroquine) and IR signaling inhibitor (linsitinib). Each inhibitor was used at 30 µM concentration for 24 h (*n* = 3).

Reduced viability was further confirmed based on a reduced metabolic activity in Nrp2^−/−^ CRC organoids after the combined inhibition of IR signaling and early autophagy (Figure S5b). Moreover, the combined inhibition of IR signaling and early autophagy led to tight junction destabilization demonstrated by reduced TJP2 and OCLN levels in Nrp2^−/−^ CRC organoids (Figure 7a), which was not observed at the combined inhibition of IR signaling and late autophagy (Figure 7b).

### 3.6. Nrp2 Expression Is Associated with a Mesenchymal Molecular Subtype of Human CRC and Inversely Correlated with Autophagy Marker Expression

**Figure 8:**
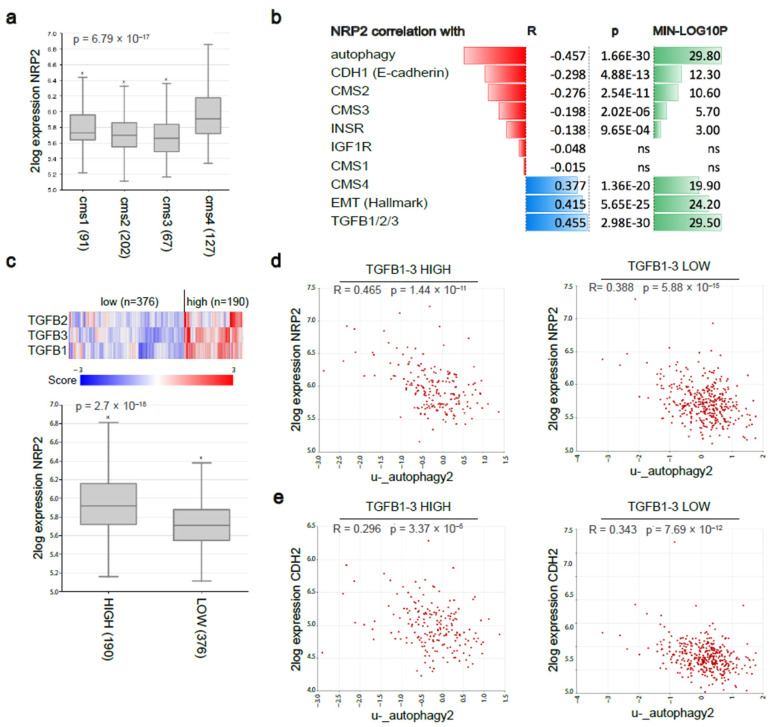
Nrp2 expression and gene-signature correlation analyzes in human CRC. (**a**) RNAseq analysis of Nrp2 RNA expression in the four consensus molecular subtypes in a large composite cohort of primary CRC (*n* = 3232) [5]. (**b**) Nrp2 gene-gene and gene-signature correlation analyzes in the human CRC tumor cohort. (**c**) Human CRC tumor subgrouping into TGFB1-3 HIGH (*n* = 190) and LOW (*n* = 376) subgroups using K-means clustering. Nrp2 expression in TGFB1-3 HIGH and LOW subgroups. Gene-signature correlation analysis between (**d**) Nrp2 expression and autophagy, (**e**) CDH2 expression and autophagy, in TGFB1-3 HIGH and LOW subgroups of human CRC.

We previously reported that lymphangiogenic marker expression, including Nrp2, was predictive of an aggressive mesenchymal molecular subtype and poor prognosis in both primary CRC and CRLM [6]. For additional evidence, we explored a publicly available CRC dataset Tumor Colon CIT (Combat)-Marisa-566-rma-u133p2 in the R2: Genomics Analysis and Visualization Platform. Based on high-density transcriptome data obtained by RNA sequencing of 566 tumor tissues, CRC tumors were classified into distinct molecular subtypes. Nrp2 was expressed at the highest levels in the CMS4 subtype of CRC (Figure 8a). In line with our findings in mouse CRC organoids, a strong positive correlation was found between Nrp2 expression and signatures identifying the mesenchymal CRC subtype (CMS4), EMT, and TGFβ1-3. In addition, Nrp2 expression shows a strong negative correlation with gene signatures identifying autophagy, ‘epithelial’ CRC subtypes (CMS2, CMS3), the junction marker E-cadherin (CDH1), and the insulin receptor (INSR). No significant correlation was found with the expression of IGF1R (Figure 8b). To explore the TGFβ-dependency of Nrp2 knockout effects, we generated LOW and HIGH TGFB1-3 subgroups by performing K-means clustering using a TGFβ ligand signature (TGFB1, TGFB2, TGFB3). Nrp2 expression was significantly higher in the TGFB1-3-HIGH subgroup (Figure 8c). Interestingly, the negative correlation between Nrp2 expression and autophagy was considerably stronger in the TGFB1-3-HIGH subgroup (R = −0.465) than in the TGFB1-3-LOW subgroup (R = −0.388) (Figure 8d). This could indeed point to a TGFβ-dependency on the effects of Nrp2 knockout. This was not observed for the negative correlation between CDH2 (a mesenchymal marker) and autophagy (Figure 8e).

## 4. Discussion

In this study, we demonstrate that Nrp2 deletion in mesenchymal-like CRC organoids results in a drastically altered phenotype characterized by MET and an acquired vulnerability to the inhibition of IR signaling and autophagy. Given that both IR signaling and autophagy are dynamic processes susceptible to the influence of nutrient availability and cellular stress, our data are in concert with a significant and complex interaction between these two major regulatory (anabolic and catabolic) mechanisms [46,47,48]. IR signaling is known to contribute to CRC development and therapy resistance [49,50]. Autophagy plays a tumor- and metastasis-promoting role in established cancers, as it provides building blocks for anabolic processes through the intracellular recycling of macromolecules [51,52,53]. We demonstrate that combined inhibition of both IR signaling and early autophagy significantly reduces CRC organoid viability in the absence of Nrp2. Thus, Nrp2 loss creates a previously non-existing vulnerability that can be therapeutically exploited. Furthermore, the loss of Nrp2 expression in murine mesenchymal-like CRC organoids results in a less aggressive, non-mesenchymal phenotype, which is not characterized by accumulation of stem-like phenotype. In particular, the adult Lgr5+ intestinal stem cell signature is significantly reduced in Nrp2^−/−^ organoids, while the fetal intestinal stem cell signature remains unaffected. These data reinforce the link between cancer stemness (reflected by the Lgr5 signature) and EMT in colon cancer and identify Nrp2 as an important factor in maintaining this phenotype.

We previously reported that Nrp2 expression identifies aggressive mesenchymal molecular subtypes in primary and metastatic CRC [6]. Nrp2^−/−^ CRC organoids demonstrate a high expression of PARD3 and OCLN in tight junctions, which are known to be responsible for establishing and maintaining epithelial cell polarity [54]. The loss of cell-cell junctions and apical-basal polarity is required for EMT [55], which is an energy-demanding process that requires metabolic reprogramming [56]. Autophagy has been reported to stabilize intestinal epithelial tight junctions under nutrient starvation conditions [57]. Hence, increased autophagy upon Nrp2 loss could potentially contribute to tight junction stabilization and EMT inhibition. MET transition, following the Nrp2 knockout in CRC organoids, is featured by a reduction of EMT drivers, TGFβ signaling, and stromal infiltration. Our work further extends these observations in human CRC by demonstrating a strong negative correlation between Nrp2 expression and gene signatures identifying autophagy, ‘epithelial’ CRC subtypes (CMS2, CMS3), the junction marker E-cadherin (CDH1), and the insulin receptor (INSR). A strong positive correlation is found between Nrp2 expression and signatures identifying the mesenchymal CRC subtype (CMS4), EMT, and TGFβ1-3. Possible TGFβ-dependency of Nrp2 knockout effects could be indicated, as Nrp2^−/−^ mouse CRC organoids demonstrate a significant reduction in SMAD2 phosphorylation. Furthermore, Nrp2 expression is significantly higher in the TGFB1-3-HIGH subgroup of human CRC.

Taken together, our findings point to a complex interaction between Nrp2 and alternative pro-survival mechanisms in aggressive CRC, which could be exploited in cancer treatment. Furthermore, ongoing clinical trials investigate autophagy inhibition in combination with cytotoxic chemotherapies and targeted agents in various cancers, including CRC [58]. In addition to its pro-tumor role, autophagy can suppress tumor initiation by limiting inflammation, tissue damage, and genome instability [59]. In vivo studies and clinical trials with autophagy inhibition, especially in combination therapy, report possible cytotoxicity issues [58,60]. Defining the context-specific role of autophagy in cancer will be important to guide autophagy-based therapeutic interventions. The possibility to establish tumor-derived 3D organoids from various types and phases of cancer progression would enable exploration of these mechanisms in an in vitro environment closely mimicking the original tumor tissue. Organoid models could be utilized to identify the susceptible cancer subtypes based on mutational background, metabolic alterations, and to improve the therapeutic window of combination therapies through autophagy inhibition.

## 5. Conclusions

We utilized murine mesenchymal-like CRC organoids to induce CRISPR-Cas9-mediated Nrp2 knockout. Nrp2 depletion resulted in a less invasive phenotype in CRC organoids, demonstrated by MET and cell-cell junction stabilization. In addition, transcriptome profiling and signaling pathway analysis revealed de novo acquired dependency of Nrp2-knockout organoids on survival mechanisms, such as IR signaling and autophagy. To note, the acquired phenotype in CRC organoids upon Nrp2-knockout was preserved in subcutaneous tumors and was extended in human CRC tumors.

Tumor-derived organoids harbor the genetic heterogeneity and phenotypic characteristics of the initial cancer tissue. Thus, organoid models established from various stages of CRC progression, with distinct mutational and phenotypic characteristics, could serve as a robust model to study underlying mechanisms and cytotoxic effects of therapeutic modalities.

## Data Availability

Data are contained within this article and the Supplementary Material. RNA sequencing data are available at the R2: Genomics Analysis and Visualization Platform (http://r2.amc.nl (accessed on 24 May 2019)).

## Supplementary Materials

The following are available online at https://www.mdpi.com/article/10.3390/cancers14030671/s1, Table S1: Nrp2 single guide RNA (sgRNA) sequences; Table S2: Nrp2 oligo sequences to clone into LentiCRISPRv2 sgRNA scaffold; Table S3: Nrp2 genomic DNA primer sequences for five Nrp2 CRISPR-Cas9 plasmids; Table S4: Murine organoid culture medium composition; Table S5: The list of antibodies; Figure S1: Generation and characterization of Nrp2^−/−^ CRC organoids; Figure S2: Increased receptor tyrosine kinase (RTK) and tight junction protein (TJP) RNA expression in Nrp2^−/−^ CRC organoids; Figure S3: Increased tight junction protein expression in Nrp2^−/−^ CRC organoids; Figure S4: Increased autophagic flux in Nrp2^−/−^ CRC organoids; Figure S5: Decreased viability and regeneration in Nrp2^−/−^ CRC organoids; Figure S6: Immunoblots used to generate Figure 4b; Figure S7: Immunoblots used to generate Figure 5b; Figure S8: Immunoblots used to generate Figure S1; Figure S9: Immunoblots used to generate Figure S3. The whole western blot figures can be found in the supplementary materials. File: Nrp2^−/−^ CRC Organoid Generation using CRISPR-Cas9 technology; Flow Cytometry: Cell Cycle Analysis via DNA Content Quantification; Organoid Viability assay; Clone Forming assay.
