# Supplementary material for: Loss of Neuropilin-2 in Murine Mesenchymal-like Colon Cancer Organoids Causes Mesenchymal-to-Epithelial Transition and an Acquired Dependency on Insulin-Receptor Signaling and Autophagy
Source: Cancers (Basel). 2022 Jan 28;14(3):671. doi: 10.3390/cancers14030671 (PMC8833430; doi:10.3390/cancers14030671)
Supplement: Supplementary file 1 [file cancers-14-00671-s001.zip › Figure S6 (Blot 1).pdf]

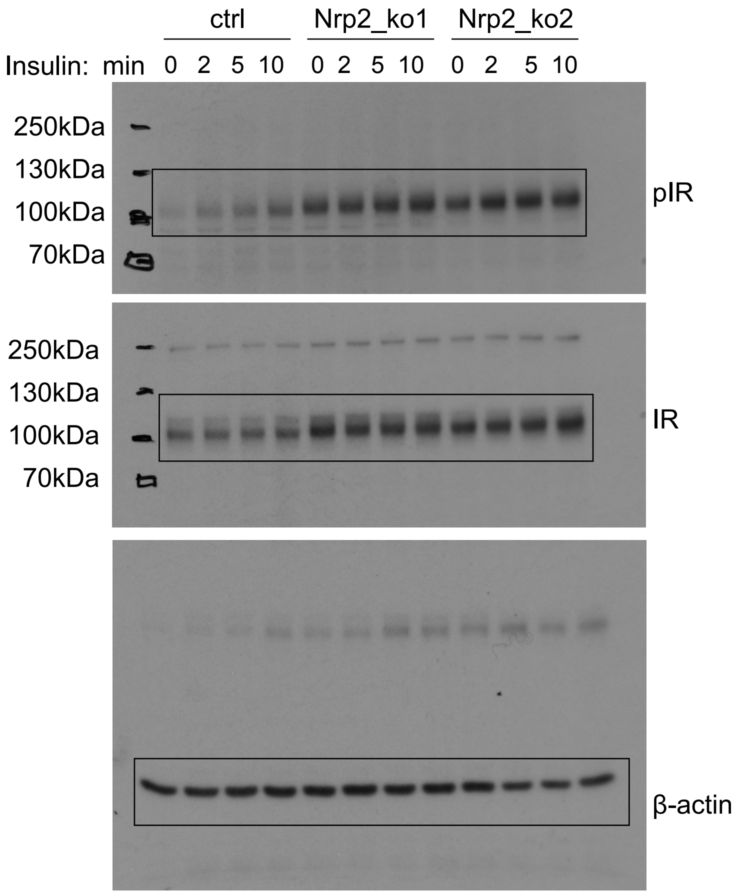

Blot1. Immunoblots used to generate Figure 4b. Representative blots from protein analysis of insulin-stimulated CRC organoids. Bands used for figure generation are highlighted.
