# Supplementary material for: Loss of Neuropilin-2 in Murine Mesenchymal-like Colon Cancer Organoids Causes Mesenchymal-to-Epithelial Transition and an Acquired Dependency on Insulin-Receptor Signaling and Autophagy
Source: Cancers (Basel). 2022 Jan 28;14(3):671. doi: 10.3390/cancers14030671 (PMC8833430; doi:10.3390/cancers14030671)
Supplement: Supplementary file 1 [file cancers-14-00671-s001.zip › Supplementary file.pdf]

Supplementary file

Loss of Neuropilin-2 in Murine Mesenchymal-like Colon Cancer Organoids Causes Mesenchymal-to-Epithelial Transition and an Acquired Dependency on Insulin-Receptor Signaling and Autophagy

Susanna Poghosyan, Nicola Frenkel, Aristeidis Lentzas, Jamila Laoukili, Inne Borel Rinkes, Onno Kranenburg, Jeroen Hagendoorn

$Nrp2^{-/-}$ CRC Organoid Generation using CRISPR-Cas9 technology

CRC organoids were generated as described in Materials and Methods, section 2.1. Nrp2 expression was assessed in the organoids using western blot analysis (data not shown). Two CRC organoid lines with the highest Nrp2 expression were chosen to induce Nrp2 knockout.

To introduce Nrp2 knock out in mouse CRC organoids lentiviral LentiCRISPRv2 (Addgene, 52961) one vector system was used. LentiCRISPRv2 contains two expression cassettes, hSpCas9 and the chimeric guide RNA. First, ten single guide RNA (sgRNA) oligos consisting of 20 base pairs (bp) were designed based on the mouse Nrp2 target DNA sequence (ENSMUSG00000025969), exons 1-4 and 6. Cas9 target design tool by Zhang Lab GeCKo was used (https://zlab.bio/guide-design-resources). All sgRNA sequences (Table S1) were flanked by 3bp *N*GG proto-spacer adjacent motif (PAM) sequence on the 3' end of the target Nrp2 DNA sequence, required for Cas9 nuclease binding and activity. To clone the guide sequence into the lentiviral vector sgRNA scaffold, forward and reverse oligo sequences were designed (Table S2), which were mixed with 1x T4 DNA ligase buffer (NEB) with ATP (B0202S), 10U/µl 2500U T4 polynucleotide kinase (PNK) (Thermofisher, EK0032). In total of 50µg plasmid mix (in 10µl volume) was phosphorylated and annealed at 37°C 60min, 95°C 5min, 14 cycles of 95°C 1min (5°C increment), 10°C incline using thermocycler (BioRad). A pair of annealed oligos was cloned into the pre-cut and dephosphorylated LentiCRISPRv2 vector. The ligation mix containing lentiviral vector, annealed oligo pair, 1x T4 DNA ligase buffer (NEB) with ATP (B0202S), T4 DNA ligase and water was incubated overnight in 16°C water bath.

Uncut LentiCRISPRv2 plasmid was used for the control CRC organoid generation. Uncut LentiCRISPRv2 plasmid and Nrp2 gRNA sequence-containing lentiviral plasmids were transformed into competent E.Coli. For each plasmid, ten ampicillin resistant single colonies were picked and amplified in miniprep and then maxiprep. Nrp2 gRNA incorporation into the lentiviral plasmid was confirmed with Sanger sequencing using U6 promoter primer (Macrogen).

The control and five different gRNA-containing plasmids were selected to transfect into 293T cells using calcium phosphate precipitation method [1]. In total of 30µg plasmid was used for each transfection reaction; 15µg Nrp2 CRISPR-Cas9 lentiviral plasmid, 7.5µg VSV-G and 7.5µg Pax-2 packaging plasmids. Two days after the transfection, lentivirus containing 293T medium was used to transduce CRC organoids. Briefly, three days old organoids were dissociated into single cells (described in Materials and Methods, section 2.1). Next, cells were washed using cold PBS and sterile filtered with 40µm filter to get rid of the cell clumps. Cell suspension was centrifuged at 1800rpm for 5min at room temperature. Cell pellet was resuspended in lentivirus containing medium from 293T cells, supplemented with 6µg/ml polybrene, 10µM Rho inhibitor (y27632), 0.5nM NAC (N-acetylcystein), and plated in ultralow adherent plates as a suspension culture. The next day, cell suspension was harvested and plated in adherent plates in matrigel for organoid growth. Organoid culture medium was supplemented with 0.5µg/ml puromycin to select the cells with incorporated lentiviral plasmid. Nrp2 knockout by five different gRNA containing CRISPR-Cas9 plasmids in CRC organoids was assessed with western blotting. Organoids generated with two different gRNA-containing plasmids; mNrp2-1, mNrp2-2, demonstrated a significant loss of Nrp2 expression. Nrp2 knockout in these organoids was further confirmed with DNA mutational analyses and immunofluorescence (Figure S1).

Nrp2 DNA mutational analysis in CRC organoids was performed using TIDE (Tracking of Indels by Decomposition) [2]. Briefly, genomic DNA was isolated from CRC organoids through lysing them in Direct PCR Lysis Buffer Reagent and proteinase K (0.5mg/mL) mix at 55°C constant agitation (500

RPM) overnight, followed by proteinase K deactivation at 85°C for 45min. The genomic DNA (<250ng) at the targeted region of Nrp2 was amplified using 0.5μM primers (Table S3) 200-300 bp upstream (Forward) and downstream (Reverse) of the gRNA sequence. Phusion High-Fidelity DNA Polymerase reaction with 98°C 30sec initial denaturation, 35 cycles of 98°C 10sec, 64°C 30sec, 72°C 30sec, and final extension of 72°C 7min was performed. Following the gel-electrophoresis, amplified DNA products were isolated using QIAquick Gel Extraction kit (Qiagen) and sequenced with forward primers using Sanger sequencing (Macrogen). Insertions and deletions were detected and visualized using Inference of CRISPR Edits (ICE) tool from Synthego, by aligning the control, Nrp2 expressing CRC organoid genomic DNA sequence with Nrp2$^{-/-}$ CRC organoid DNA sequence. Five nucleotide deletion was detected in both Nrp2$^{-/-}$ CRC organoid lines. No indels detected in the control CRC organoids (Figure S1b).

Two Nrp2$^{-/-}$ CRC organoid lines were further characterized for Nrp2 depletion. Principal component analysis (PCA) for RNAseq data showed distinct separation between control and Nrp2$^{-/-}$ CRC organoids (Figure S1c). Cell cycle analysis by quantification of DNA content (propidium iodide) showed increased cell count in G2 phase of the cell cycle in Nrp2$^{-/-}$ CRC organoids (Figure S1d).

References

1. Kwon, M.; Firestein, B.L. DNA transfection: calcium phosphate method. *Methods in molecular biology (Clifton, N.J.)* **2013**, *1018*, 107-110, doi:10.1007/978-1-62703-444-9_10.

2. Brinkman, E.K.; Chen, T.; Amendola, M.; van Steensel, B. Easy quantitative assessment of genome editing by sequence trace decomposition. *Nucleic Acids Research* **2014**, *42*, e168-e168, doi:10.1093/nar/gku936.

Flow Cytometry: Cell Cycle Analysis via DNA Content Quantification

Organoids were dissociated into single cells with TrypLE Express (Gibco, 12604021) for 5min at 37°C water bath. Next, cells were washed using cold PBS and sterile filtered with 40μm filter to get rid of the cell clumps. Cell suspension was centrifuged at 1800rpm for 5min at room temperature. Cell fixation was done with 70% ethanol, which was added to the cell pellet dropwise while vortexing to prevent cell clumping. Then cells were washed twice with PBS and resuspended in flow cytometry buffer consisting of 1x PBS 1% BSA and 2.5mM EDTA. To obtain pure DNA content, cell suspension was treated with 100μg/ml RNase. For DNA content analysis cell suspension was stained with 1:500 propidium iodide for 30min at room temperature, in the dark. Data were acquired using BD FACSCelesta (BD Bioscience) and analyzed using BD FACSDiva software version 8.0. Cell debires. Doublets were excluded and 10.000 events were measured.

Organoid Viability assay

Three days old organoids were harvested using 2mg/ml Dispase II (Gibco, 17105041) for 15min at 37°C. Organoids were counted and seeded at 1000 organoids per 100μl well density in 96-well plate. First, 40μl matrigel was dispensed in wells and incubated 30min at 37°C. Next, organoids were seeded in matrigel-coated wells. After 2h incubation at 37°C, organoids were treated with IR signaling inhibitor (Linsitinib, OSI-906, Selleckchem, S1091) and early autophagy inhibitor (Spautin-1, Sigma, SMLD440). Organoid viability was assessed using CellTiter-Glo 3D Cell Viability assay according to the manufacturer's instructions (Promega, G9683). Relative luminescence signal was quantified relative to the untreated control.

Clone Forming assay

Three days old organoids were treated with 2mg/ml Dispase II (Gibco, 17105041) for 15min at 37°C to disrupt matrigel droplets. Afterwards, organoids were dissociated into single cells using TrypLE Express (Gibco, 12604021) for 5min in 37°C water bath. Single cells were counted and cell suspension was prepared with 1:1 ratio of medium and matrigel. 1000 single cells in 100µl volume were plated in a well of 6-well plate. First, pipet cell suspension in a doughnut shape around the well and then spread towards the center of the well with circular moves. Let cell-matrigel suspension solidify at 37°C, and add cell culture medium into the well and incubate at 37°C. Medium was refreshed twice a week. Each condition was seeded in triplicates per assay. Clones were counted two weeks after the cell seeding, and quantified relative to the control.
