# Supplementary material for: Loss of Neuropilin-2 in Murine Mesenchymal-like Colon Cancer Organoids Causes Mesenchymal-to-Epithelial Transition and an Acquired Dependency on Insulin-Receptor Signaling and Autophagy
Source: Cancers (Basel). 2022 Jan 28;14(3):671. doi: 10.3390/cancers14030671 (PMC8833430; doi:10.3390/cancers14030671)
Supplement: Supplementary file 1 [file cancers-14-00671-s001.zip › Supplementary material.pdf]

Supplement

# Loss of Neuropilin-2 in Murine Mesenchymal-like Colon Cancer Organoids Causes Mesenchymal-to-Epithelial Transition and an Acquired Dependency on Insulin-Receptor Signaling and Autophagy

**Susanna Poghosyan, Nicola Frenkel, Aristeidis Lentzas, Jamila Laoukili, Inne Borel Rinkes, Onno Kranenburg, Jeroen Hagendoorn**

**Table S1.** Nrp2 single guide RNA (sgRNA) sequences

| sgRNA | Exon | Strand | Sequence 5′- 3′ |
|---|---|---|---|
| mNrp2- 1 | 1 | Forward | TGGATATGTTTCCTCTTACC |
| mNrp2- 2 | | Forward | GGATATGTTTCCTCTTACCT |
| mNrp2- 3 | 2 | Reverse | GGAATTCAGCCGACCTCCGC |
| mNrp2- 4 | | Reverse | TTCAGCCGACCTCCGCAGGG |
| mNrp2- 5 | 3 | Forward | CACCTCAGACTACGCCCGGC |
| mNrp2- 6 | | Forward | AGTTCACCTCAGACTACGCC |
| mNrp2- 7 | 4 | Forward | GCATGACCCTCTACAAGTGG |
| mNrp2- 8 | | Forward | GAGATTCAATGGTCCCATTG |
| mNrp2- 9 | 6 | Forward | CAATGGCTGGACACCCAATT |
| mNrp2- 10 | | Forward | CCATGGAGCCGGCTCTGTTG |

**Table S2.** Nrp2 oligo sequences to clone into LentiCRISPRv2 sgRNA scaffold

| sgRNA oligo | Sequence 5′- 3′ |
|---|---|
| mNrp2- 1F<br>mNrp2- 1R | CACCGTGGATATGTTTCCTCTTACC<br>AAACGGTAAGAGGAAACATATCCAC |
| mNrp2- 2F<br>mNrp2- 2R | CACCGGGATATGTTTCCTCTTACCT<br>AAACAGGTAAGAGGAAACATATCCC |
| mNrp2- 3F<br>mNrp2- 3R | CACCGGGAATTCAGCCGACCTCCGC<br>AAACGCGGAGGTCGGCTGAATTCCC |
| mNrp2- 4F<br>mNrp2- 4R | CACCGTTCAGCCGACCTCCGCAGGG<br>AAACCCCTGCGGAGGTCGGCTGAAC |
| mNrp2- 5F<br>mNrp2- 5R | CACCGCACCTCAGACTACGCCCGGC<br>AAACGCCGGGCGTAGTCTGAGGTGC |
| mNrp2- 6F<br>mNrp2- 6R | CACCGAGTTCACCTCAGACTACGCC<br>AAACGGCGTAGTCTGAGGTGAACTC |
| mNrp2- 7F<br>mNrp2- 7R | CACCGGCATGACCCTCTACAAGTGG<br>AAACCCACTTGTAGAGGGTCATGCC |
| mNrp2- 8F<br>mNrp2- 8R | CACCGGAGATTCAATGGTCCCATTG<br>AAACCAATGGGACCATTGAATCTCC |
| mNrp2- 9F<br>mNrp2- 9R | CACCGCAATGGCTGGACACCCAATT<br>AAACAATTGGGTGTCCAGCCATTGC |
| mNrp2- 10F<br>mNrp2- 10R | CACCGCCATGGAGCCGGCTCTGTTG<br>AAACCAACAGAGCCGGCTCCATGGC |

**Table S3.** Nrp2 genomic DNA primer[1] sequences for five Nrp2 CRISPR-Cas9 plasmids

| Primer | Sequence 5′- 3′ |
|---|---|
| NRP2_1.1_F<br>NRP2_1.1_R | GGCTGGGAGACACCGAATAA<br>TAGCAGCTGTGGAAATGGGG |
| NRP2_1.1_1F<br>NRP2_1.1_1R | GTCGGCACCACAAAACCAC<br>TTTTGTAGTCCAGCCCCTGC |
| NRP2_5.1_F<br>NRP2_5.1_R | ATGGTGCATCTGGCATCTGT<br>GGGGCACCTTGAGTTCTGTT |
| NRP2_5.1_1F<br>NRP2_5.1_1R | TGGTGCATCTGGCATCTGTAG<br>GGCAGGGCTCTCTCCAATAG |
| NRP2_3.1_F<br>NRP2_3.1_R | ATTTATGAGGGCTTGAAACGTGC<br>AGCCTTGGGTGATGTACCTTTT |
| NRP2_3.1_1F<br>NRP2_3.1_1R | TCTCTGTACCTCCACCCCAC<br>TGATGATAGCAGCTAACTTAACACA |
| NRP2_6.1_F<br>NRP2_6.1_R | AGCATCTGCTTCCACAGTCC<br>GAGCCCATCCCCAACAAACT |
| NRP2_6.1_1F<br>NRP2_6.1_1R | TCCAAGGCAGGCCATCATTT<br>CAGAGCCCATCCCCAACAAA |
| NRP2_2.2_F<br>NRP2_2.2_R | AACGATGCTCCTGCTCTTACG<br>TGGTAACATGGACTGCCAAGG |
| NRP2_2.2_1F<br>NRP2_2.2_1R | GATGCTCCTGCTCTTACGTG<br>GTAACATGGACTGCCAAGGG |

[1] Primers were designed based on mouse Nrp2 NCBI reference sequence NC_000067.6 using NCBI primer design tool. Primer quality and annealing temperatures were checked using NCBI and NEB Tm calculator tools.

**Table S4.** Murine organoid culture medium composition

| Component | Supplier (catalog number) | Concentration |
|---|---|---|
| Advanced DMEM/F12 basal medium | Gibco (12634-010) | 1x (500ml total) |
| HEPES buffer | Lonza (BE17-737E) | 10mM |
| Penicillin-Streptomycin | Gibco (15070-063) | 50U/ml |
| GlutaMAX | Gibco (35050-038) | 2mM |
| Noggin conditioned medium | 293T-mNoggin-Fc cell line | 100ng/ml |
| B27, serum free | Invitrogen (17504-044) | 1x |
| Recombinant murine FGF-basic | PeproTech (450-33) | 10ng/ml |

**Table S5.** The list of antibodies

| Assay | Antibody (clone) | Supplier (catalog number) | Dilution |
|---|---|---|---|
| Western blot | TJP2 | Invitrogen (71-1400) | 1:150 |
| | LC3II (0510) | Nanotools (0231-100/LC3-5F10) | 1:1000 |
| | Nrp2 (D39A5) | Cell Signaling (3366) | 1:1000 |
| | IR-β (4B8) | Cell signaling (3025) | 1:1000 |
| | phospho IGFIRβ-IRβ | Cell signaling (3021) | 1:1000 |
| | PI3 kinase p85 | Cell signaling (4292) | 1:1000 |
| | phospho PI3 kinase p85/p55 | Cell signaling (4228S) | 1:1000 |
| | p44/42 MAPK (Erk1/2) (137F5) | Cell signaling (4695) | 1:1000 |
| | phospho P44/42 MAPK (Erk1/2) | Cell signaling (9101) | 1:1000 |
| | PARD3/ Par3 | Novus Biologicals (NBP1-88861) | 1:150 |
| | Occludin (OCLN) | Thermofisher (40-4700) | 1:250 |
| | β-actin (AC-15) | Novus Biologicals (NB600-501) | 1:10000 |
| | α-tubulin (B-5-1-2) | Sigma (T5168) | 1:10000 |
| | Goat anti-rabbit IgG HRP | Dako (P0448) | 1:1000 |
| | Goat anti-mouse IgG HRP | Dako (P0447) | 1:2000 |
| IHC | ZEB1 (2A8A6) | Novus Biologicals (NBP2-23484SS) | 1:250 |
| | Slug | Novus Biologicals (NBP2-27182SS) | 1:100 |
| | TJP2 | Novus Biologicals (NBP1-86850) | 1:100 |
| | LC3II (0510) | Nanotools (0231-100/LC3-5F10) | 1:100 |
| | IR-β (E9L5V) | Cell signaling (23413S) | 1:50 |
| | PARD3/ Par3 | Novus Biologicals (NBP1-88861) | 1:500 |
| | Occludin (OCLN) | Thermofisher (40-4700) | 1:100 |
| IF | LC3II (0510) | Nanotools (0231-100/LC3-5F10) | 1:50 |
| | p62/SQSTM1 | Novus Biologicals (NBP1-48320) | 1:200 |
| | Cleaved caspase-3 | Cell Signaling (9661S) | 1:400 |
| | PARD3/ Par3 | Novus Biologicals (NBP1-88861) | 1:50 |
| | TJP2 | Invitrogen (71-1400) | 1:300 |
| | IR-β (E9L5V) | Cell signaling (23413S) | 1:50 |
| | Nrp2 (D39A5) | Cell Signaling (3366) | 1:1000 |
| | DAPI | Sigma (D9542) | 1:1000 |
| | Phalloidin AlexaFluor488 | ThermoFisher (A12379) | 1:500 |
| | Alexa Fluor 568 | ThermoFisher (A11036) | 1:500 |

**Figure S1.** Generation and characterization of Nrp2$^{-/-}$ CRC organoids. (**a**) Schematic view of lentiviral plasmids used for Nrp2$^{-/-}$ (left) or control (right) CRC organoid generation. (**b**) Sanger sequencing data used to analyze % of DNA with insertions and/or deletions in CRC organoid genomic DNA targeted location. Inference of CRISPR Edits (ICE) tool from Synthego was used. (**c**) Principal component analysis (PCA) for RNAseq data. Samples were sequenced in three replicates. (**d**) Cell cycle analysis by quantification of DNA content (propidium iodide) in CRC organoids. (**e**) Immunofluorescence analysis for Nrp2 expression in CRC organoids. Representative organoid images are depicted, where DAPI-stained nuclei are shown in blue, and membranous Nrp2 is shown in red. Scale bars: 15μm. (**f**) Western blot analysis for Nrp2 expression in CRC organoids.

**Figure S2.** Increased receptor tyrosin kinase (RTK) and tight junction protein (TJP) RNA expression in Nrp2$^{-/-}$ CRC organoids. (**a**) RTK and (**b**) TJP RNA expression in control and Nrp2$^{-/-}$ CRC organoids determined by RNA sequencing, represented in heatmaps. Samples were sequenced in three replicates.

**Figure S3.** Increased tight junction protein expression in Nrp2$^{-/-}$ CRC organoids. (**a**) TJP2 RNA and protein expression analysis in CRC organoids via RNAseq and western blotting, respectively. (**b**) PARD3 RNA and protein expression analysis in CRC organoids via RNAseq and western blotting, respectively.

**Figure S4.** Increased autophagic flux in Nrp2$^{-/-}$ CRC organoids. Immunofluorescence analysis of CRC organoids after treatment with an early autophagy inhibitor (Spautin-1) or late autophagy inhibitor (Chloroquine). Each inhibitor was used at 30μM concentration for 24h. Representative organoid images are depicted, where DAPI-stained nuclei are shown in blue, and p62 and LC3 puncta are shown in red. Mean fluorescence intensity was quantified relative to the control (n=5). Scale bars: 15μm. Error bars are presented as SD from the arithmetic mean. Statistical significance on graphs is indicated $p \leq 0.05$ by *, $p \leq 0.01$ by **, $p \leq 0.001$ by ***, $p \leq 0.0001$ by ****.

**Figure S5.** Decreased viability and regeneration in Nrp2$^{-/-}$ CRC organoids. (**a**) CellTiter-glo 3D viability analysis of CRC organoids after single or combination treatment with early autophagy inhibitor (Spautin-1) (30μM) and IR signaling inhibitor (Linsitinib) (5μM or 15μM) for 24h. Relative luminescence signal was quantified relative to the 100% untreated control. (**b**) Colony forming assay by CRC organoids. Number of colonies were counted two weeks after single cell seeding, and quantified relative to the control (n=3). Error bars are presented as SD from the arithmetic mean. Statistical significance on graphs is indicated $p \leq 0.05$ by *, $p \leq 0.0001$ by ****.

**Figure S6.** Immunoblots used to generate Figure 4b. Representative blots from protein analysis of insulin-stimulated CRC organoids. Bands used for figure generation are highlighted.

**Figure S7**. Immunoblots used to generate Figure 5b. Representative blots from protein analysis of the control and Nrp2$^{-/-}$ CRC organoids. Bands used for figure generation are highlighted.

**Figure S8.** Immunoblots used to generate Figure S1. Representative blots from protein analysis of the control and Nrp2$^{-/-}$ CRC organoids. Bands used for figure generation are highlighted.

**Figure S9.** Immunoblots used to generate Figure S3. Representative blots from protein analysis of control and Nrp2$^{-/-}$ CRC organoids. (**a**) TJP2 expression blot, (**b**) PARD3 expression blot. Bands used for figure generation are highlighted.
