# Supplementary figures and images for: Loss of Neuropilin-2 in Murine Mesenchymal-like Colon Cancer Organoids Causes Mesenchymal-to-Epithelial Transition and an Acquired Dependency on Insulin-Receptor Signaling and Autophagy
Source: Cancers (Basel). 2022 Jan 28;14(3):671. doi: 10.3390/cancers14030671 (PMC8833430; doi:10.3390/cancers14030671)

**a**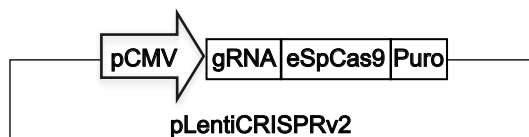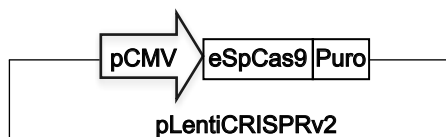**b**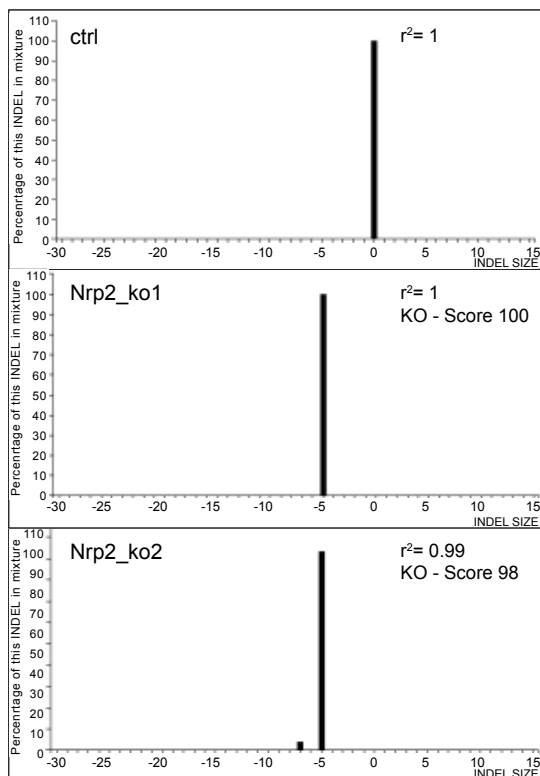**c**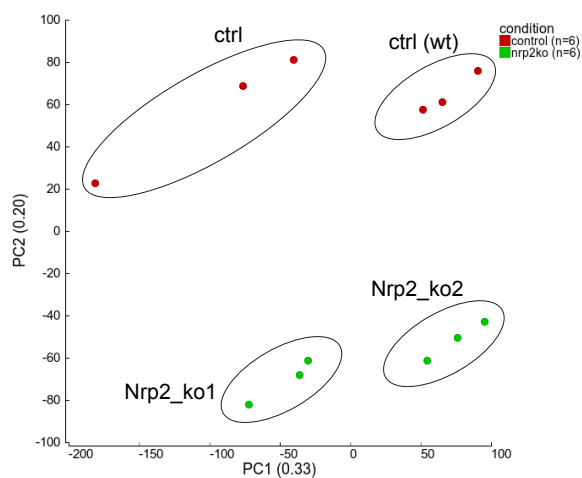**d**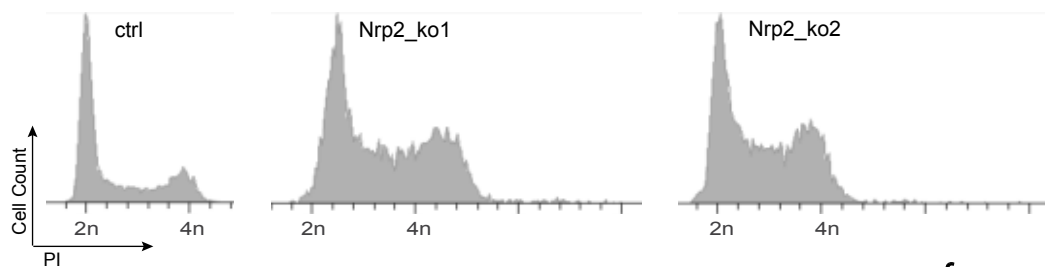**e**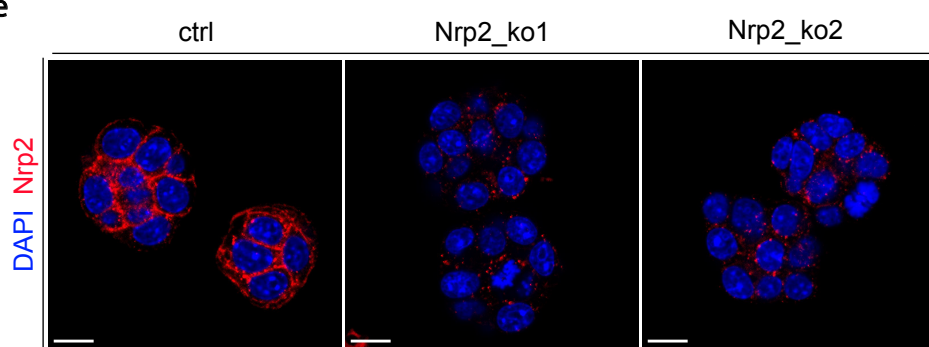**f**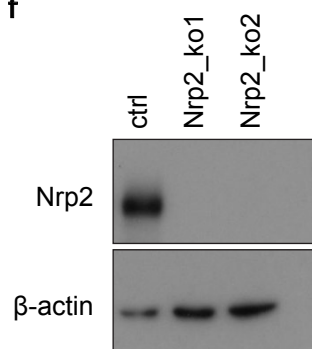

Supplement: Supplementary file 1 [file cancers-14-00671-s001.zip › Figure S1.pdf]

**a**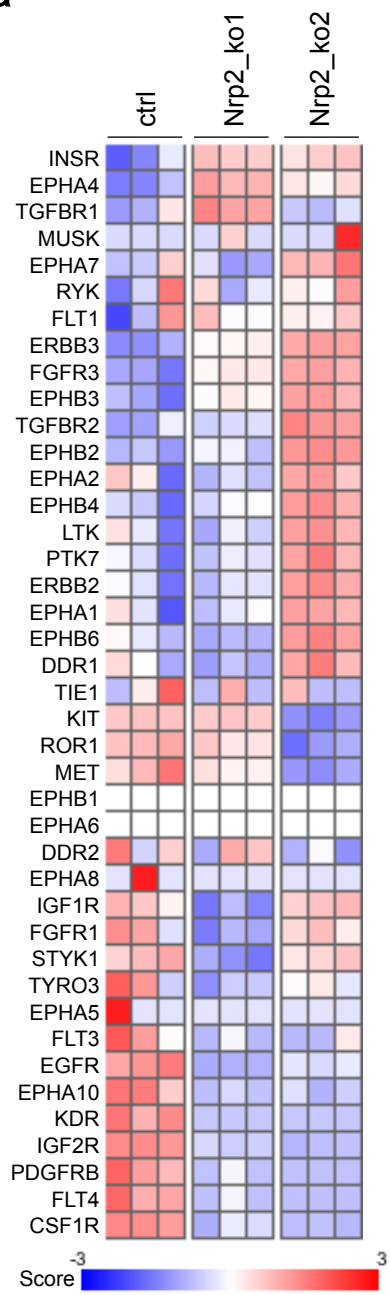**b**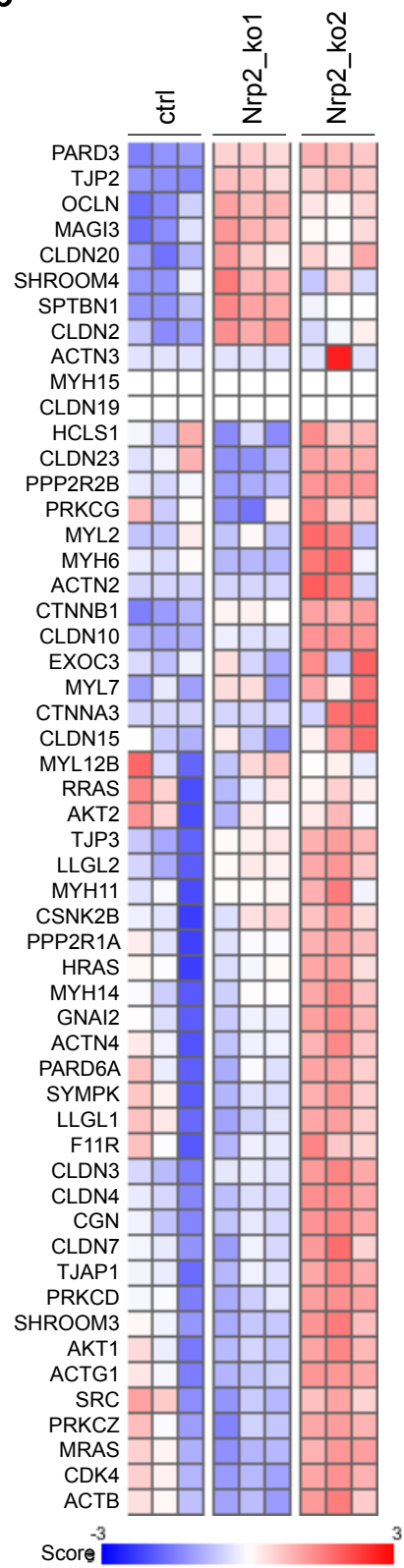

Supplement: Supplementary file 1 [file cancers-14-00671-s001.zip › Figure S2.pdf]

**a**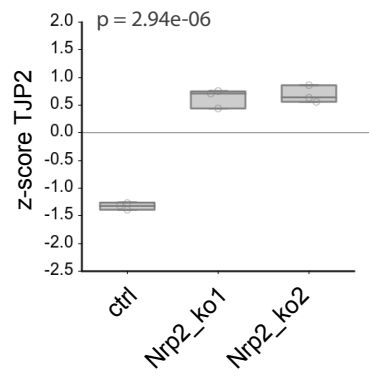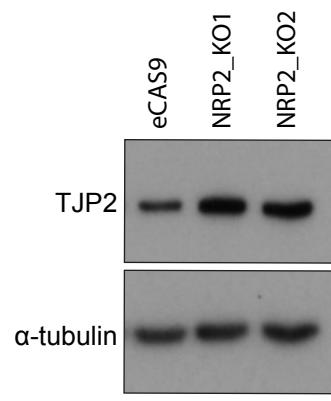**b**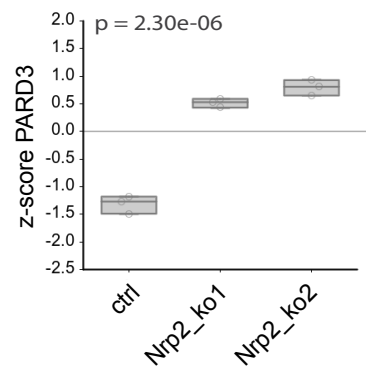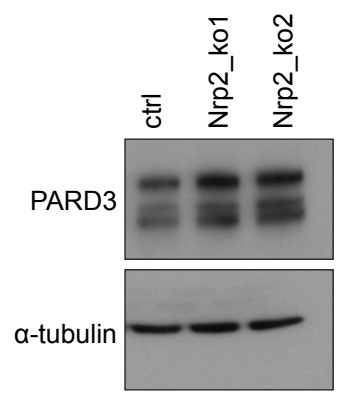

Supplement: Supplementary file 1 [file cancers-14-00671-s001.zip › Figure S3.pdf]

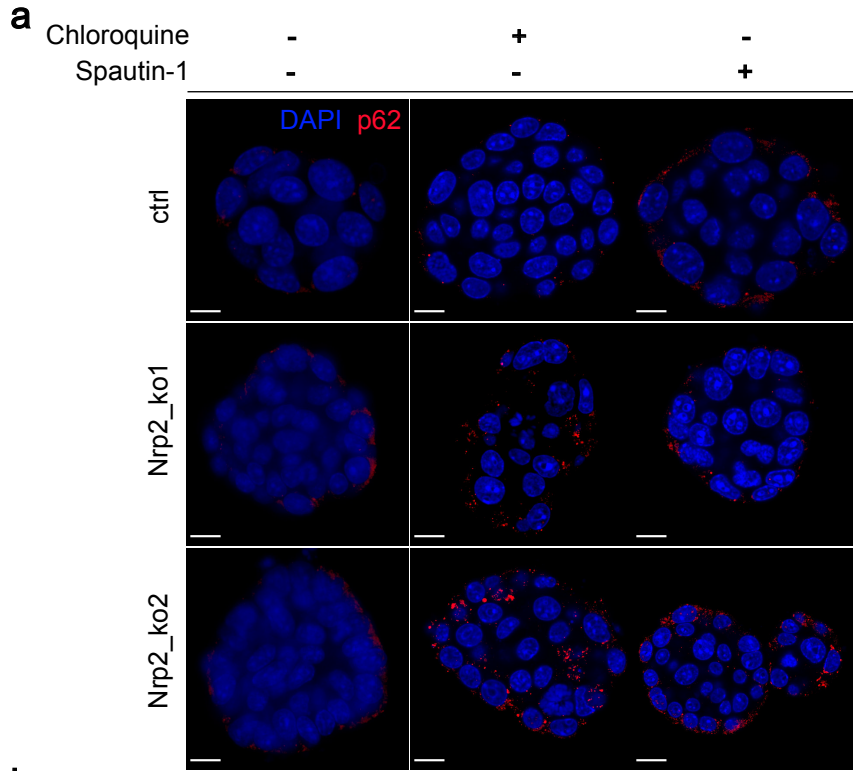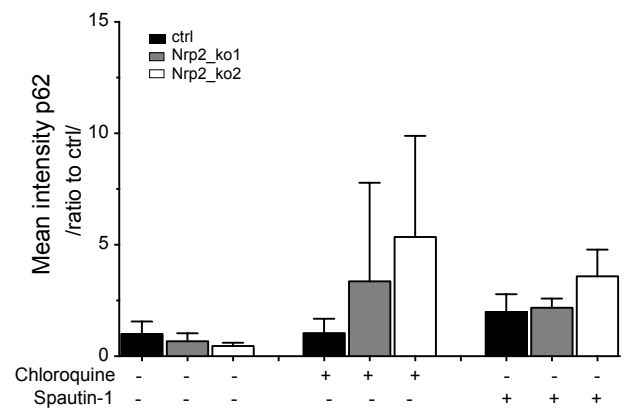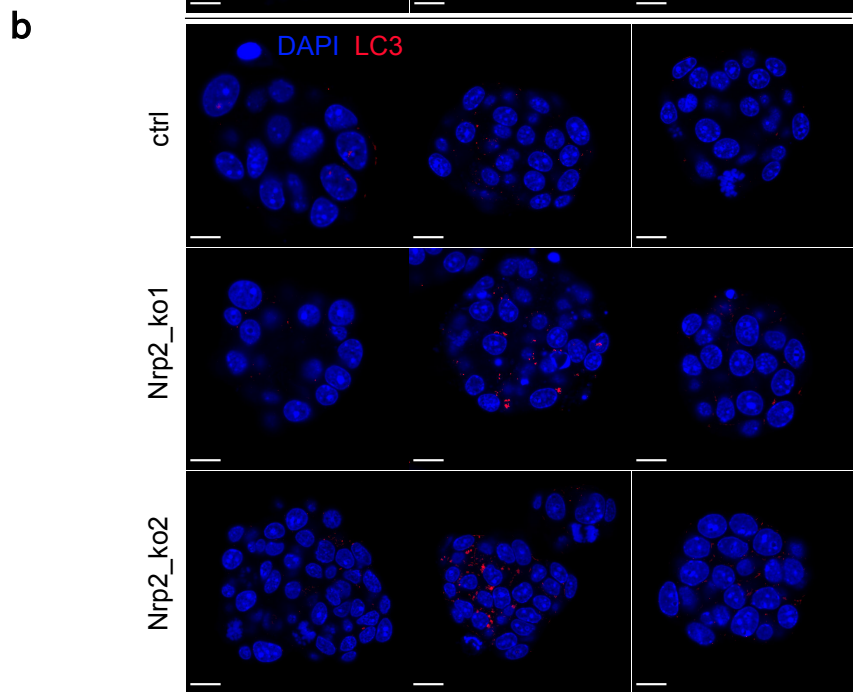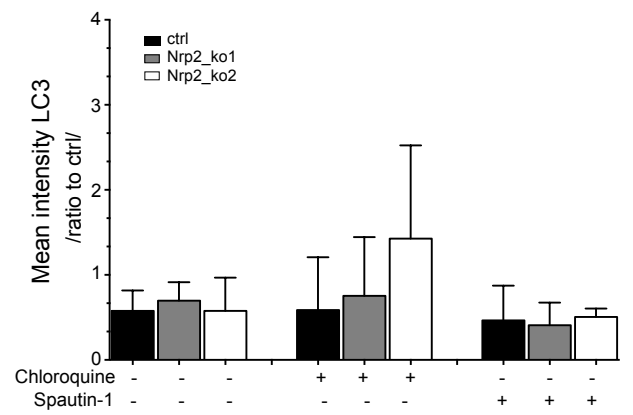

Supplement: Supplementary file 1 [file cancers-14-00671-s001.zip › Figure S4.pdf]

**a**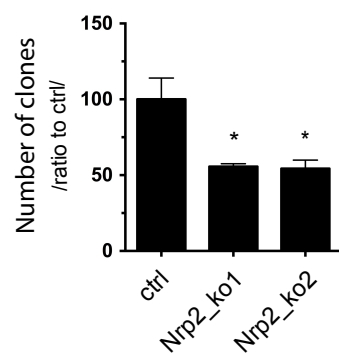**b**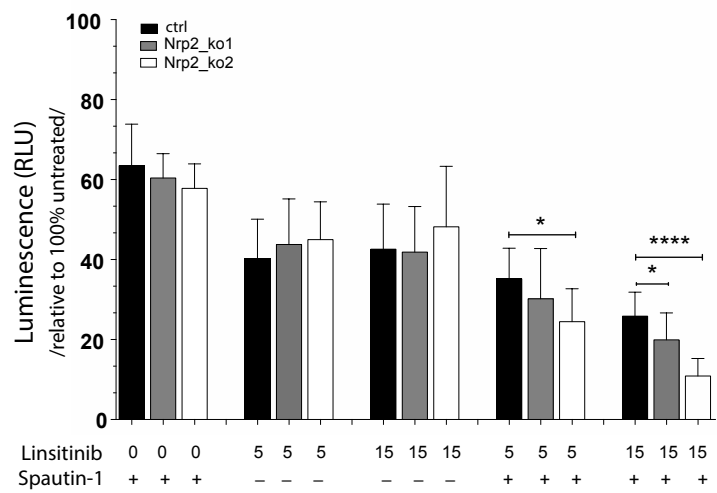

Supplement: Supplementary file 1 [file cancers-14-00671-s001.zip › Figure S5.pdf]
